# Biological Motion Perception Is Affected by Age and Cognitive Style in Children Aged 8–15

**DOI:** 10.1155/2015/594042

**Published:** 2015-03-16

**Authors:** Parisa Ghanouni, Amir Hossein Memari, Monir Shayestehfar, Pouria Moshayedi, Shahriar Gharibzadeh, Vahid Ziaee

**Affiliations:** ^1^Occupational Science and Occupational Therapy, Faculty of Medicine, University of British Columbia, Vancouver, Canada; ^2^Neuroscience Institute, Sports Medicine Research Center, Tehran University of Medical Sciences, Tehran, Iran; ^3^Department of Neurology, University of California, Los Angeles, CA, USA; ^4^Department of Biomedical Engineering, Amirkabir University of Technology, Tehran, Iran; ^5^Growth and Development Research Center, Tehran University of Medical Sciences, Tehran, Iran

## Abstract

The current paper aims to address the question of how biological motion perception in different social contexts is influenced by age or also affected by cognitive styles. We examined developmental changes of biological motion perception among 141 school children aged 8–15 using point-light displays in monadic and dyadic social contexts. Furthermore, the cognitive styles of participants were investigated using empathizing-systemizing questionnaires. Results showed that the age and empathizing ability strongly predicted improvement in action perception in both contexts. However the systemizing ability was an independent predictor of performance only in monadic contexts. Furthermore, accuracy of action perception increased significantly from 46.4% (SD = 16.1) in monadic to 62.5% (SD = 11.5) in dyadic social contexts. This study can help to identify the roles of social context in biological motion perception and shows that children with different cognitive styles may present different biological motion perception.

## 1. Introduction

Social creatures are able to understand and monitor actions in communications through perceiving intentions of others. Thus, they are sensitive and open to signals from social environment including personal level as well as situation based social cues [1, 2]. From an evolutionary perspective, wide array of social information is essential to deal with everyday life opportunities and circumstances. Among human beings, body movements or biological motions convey a part of this information which helps individuals to anticipate conditions that include a combination of social and nonsocial factors [3, 4]. Social context presents situations where a biological agent exhibits and performs a task with or without an object [5, 6]. This is in contrast with nonbiological context that implies no human being executes the behaviour. Although little is known about the development of context sensitivity in motion detection, few previous studies reported that some parts of the brain including the superior temporal sulcus (STS) and primary motor cortex show greater response to the biological than to the nonbiological motion stimuli [7, 8]. Furthermore it is proposed that differences in other factors such as character of social contexts (person-person versus person-object interactions) may contribute to differences in context utilization and mechanisms of action perception [5, 9, 10]. Recently, Clarke et al. investigated emotion perception using point-light displays (PLDs) of human movements. Their results indicate that interactive social information rather than noninteractive information may enhance emotion perception of participants [6]. From the viewpoint of developmental social neuroscience, the ability to identify actions in a dyadic context (i.e., person-person interaction) or a monadic context (i.e., person-object interaction) may be an important developmental phenomenon. With cognitive progress, children are gradually able to attribute and understand intentions behind an interaction, possibly with different patterns and paces in different social contexts (monadic versus dyadic contexts) [11].

Individuals in different age groups will recognize social meanings of behaviours differently [1, 12]. It has been demonstrated that infants have inner tendency to distinguish animate beings from objects and tend to look more at biological motions rather than nonbiological motions [13–15]. Developmental studies reveal that perceptual proficiency can be improved among children via acquiring experiences in different contexts [4, 16, 17]. Experiences particularly in visuomotor fields enable children to infer others' performance by reflecting on their own mental representations [14, 18, 19]. Also, studies have shown individual differences and possible factors that may influence perceiving social actions during development. For example, while the young children usually use a holistic approach in scanning or perceiving an interaction (monads or dyads), the older children replace this to some extent with an analytic approach [20]. Thus, it may be noted that children and adolescents use different cognitive processes for action perception in social contexts [20–22]. The fundamental issue in this case is whether the perception of an action in monadic or dyadic contexts relates to the type of information processing.

Recently it has been shown that there is a continuum of cognitive style which can result in different perception of others' mental state [23]. Based on empathizing-systemizing (*E-S*) theory, individuals with higher drive to systemize tend to pay more attention to details and achieve subtle differences (analytic approach) whereas empathizers prefer to recruit information of a context as a whole (holistic approach) [23, 24]. Now, it is established that empathizing ability is well associated with emotion recognition via biological motion movies [3]. Also, examining neural correlates indicated that STS and prefrontal cortex which are in charge of biological motion perception can also contribute to intention attribution skills (i.e., empathizing) [25, 26]. Thus, it can be hypothesized that empathizing ability is linked to intention reading and accordingly to action perception. On the other hand the role of systemizing as a competitor of empathizing ability in intention reading or action perception is not yet clear. However, social contexts may be a good opportunity for examining the theory of* E-S* that originally has been developed on autism disorder [27]. Indeed the spectrum of cognitive styles may play a critical role in the way of perceiving visual inputs, processing the information, and also perceiving the actions [28].

Based on the given background, we hypothesized that children show better performance in dyadic than monadic contexts but with different developmental patterns. We further believed that empathizing and systemizing scores would influence accuracy of action perception in social contexts. Therefore, we used PLDs of human actions in different monads and dyads to investigate the developmental trend of action perception. More precisely, we also tried to explain the effects of individuals' cognitive style on action perception in social contexts.

## 2. Methods

### 2.1. Participants

Participants for this study consisted of 141 students aged from 8 to 15 years who had normal or corrected to normal vision. They were recruited from a local school in Tehran, randomly from grades 2–9, using a probability proportional to size sampling. All of participants were naive in terms of target of study and had no previous experience in taking part in similar experiments. Written consent was obtained from either parents or participants. This study was approved by the Medical Ethics Committee of Tehran University of Medical Sciences.

### 2.2. Cognitive Measurement

To examine the empathy quotient (EQ) and systemizing quotient (SQ), we applied combined questionnaire of EQ and SQ for children <12 years old [29] while adult version questionnaires were used for adolescents (>12 years). All the questionnaires have been statistically validated and proved to be reliable in previous experiments [30]. Children's questionnaire consisted of 55 items of which 27 items represented EQ, and the remaining 28 queries stood for SQ rate. However, each adult questionnaire consists of 40 pure queries in order to estimate EQ and SQ [30, 31]. Children questionnaires were completed by parents; however those for adolescents were self-reported. There were four alternative options from strongly agree to strongly disagree for each question. Individuals were supposed to fill out questionnaires and choose the degree of their agreements as accurately as possible. Each question was scored based on the extent of agreement from score 0 to 2; then they were summed for the total score [29, 30]. The highest score for children's EQ, SQ and adolescents' EQ, SQ was 54, 56, 80, and 80, respectively, where the higher scores in each category reveal the greater tendency to that respective ability. Finally we computed a standardized score of EQ (*E*) and SQ to provide comparable scores for empathizing and systemizing measures of all participants (for more details see the statistical analysis).

### 2.3. Stimuli

There were two different sets of PLDs depicting human movements including dyads and monads from lateral view angle with a frame rate of 30 Hz. The dyads consisted of 20 types of point-light displays which represented actions with two actors in the interpersonal contexts such as “sit down,” “stand up,” “go,” and “stop” [5]. The monads also consisted of 20 types of point-light displays which showed actions with one actor such as “cycle,” “drive,” “jump,” and “walk” [32]. Each actor in monads or dyads was shown by 13 bright markers (head, shoulders, elbows, wrists, hips, knees, and feet) in a black background. To enhance the comparability of dyads with monads we included the point-lights belonging only to male actors.

During the experiments, the stimuli were shown on a 15.6-inch monitor (75 Hz refresh rate, 1,024 × 768 pixels). Children viewed the display in a quiet room from a distance of 50 cm. All the point-lights were shown in bright dots on a black background. The stimuli were viewed binocularly and always presented within a boxed area of the screen subtending a size of 9° of visual angle vertically and 11° horizontally. Duration of each display was about two seconds similar to previous studies [5, 32].

### 2.4. Procedure

Each participant did two distinct primary trials in order to get familiar with the experiment. After making sure that children got acquainted with what they should do, the main test was commenced. Individuals were invited to watch each PLD two times. Then, they were asked to provide a short description of the actions based on PLDs. Participants' responses were scored by a single rater blind to the purpose of the experiment. For each display, the rater was asked to determine whether the action had been correctly described or not. The experiment presented a series of 40 × 2 trials which were shown in a randomized order counterbalanced across participants. There was no emotional feedback on behalf of the examiner, no time constraints on giving responses, and no restrictions for mimicking the movies by participants.

### 2.5. Statistical Analysis

We calculated the percentage of accurate responses for each participant in dyads as well as monads separately. To provide comparable scores for empathizing and systemizing measures of all participants, we computed a standardized score of EQ (*E*) and SQ (*S*) by subtraction of the population mean from raw scores and divided the outcome by the maximum possible score of each questionnaire (*S* = (SQ − mean)/SQ max,* E* = (EQ − mean)/EQ max) [29]. Then, multiple regression analysis was performed to determine the independent effects of age, empathizing, and systemizing variables on performances in each social context. A paired* t*-test was conducted to examine differences in performance in dyadic versus monadic social contexts. Furthermore we examined independent variables (such as empathizing, systemizing, age, and education) that could predict mean differences of performance in dyadic versus monadic contexts using a linear regression model. The *P* values less than 0.05 were considered as the level of significance. Analyses were performed using the SPSS software version 17 (SPSS Inc., Chicago, IL, USA).

## 3. Results

Analysis showed that primary assumptions of parametric testing (i.e., normal distribution) were largely fulfilled.

**Figure 1:**
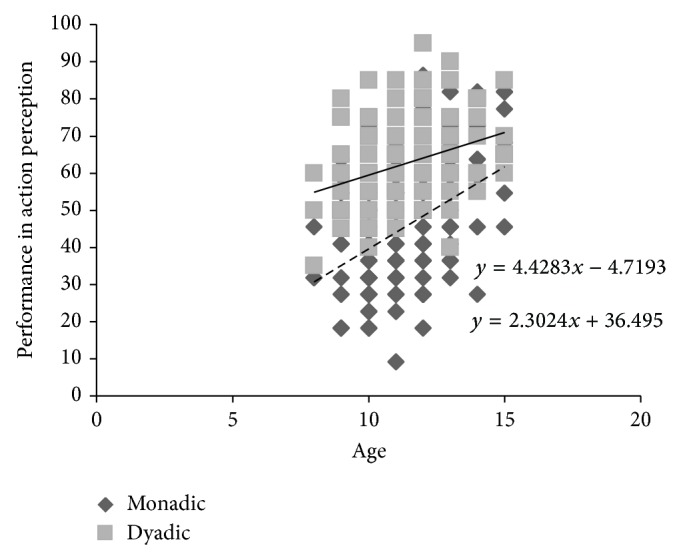
Performance in action perception in monadic and dyadic contexts across age.* Note*. Dashed line represents a regression line fit with data of monadic point lights and the solid line represents a regression line fit with data of dyadic point lights.

As can be seen from Table 1, investigating correlates of performance in dyadic and monadic social contexts, multiple regression analysis indicated that both age (*t* = 4.09, *P* < 0.001) and empathizing (*t* = 3.97, *P* < 0.001) significantly predicted performance in dyadic context (adjusted* R* ^2^ = 0.20). It was also showed that age (*t* = 6.36, *P* < 0.001), empathizing (*t* = 4.45, *P* < 0.001), and systemizing (*t* = 2.11, *P* = 0.03) were the significant predictors of action perception in monadic context (adjusted* R* ^2^ = 0.37).  Figure 1 showed association of age with action performance in each context.

Paired* t*-test was used to examine differences of action perception in two contexts (dyads versus monads). There was a significant difference in performance of participants between two contexts (*t* = 12.17, 95% CI = 13.50–18.73, *P* < 0.001). In fact, accuracy of action perception increased significantly from 46.4 (SD = 16.1%) in monadic to 62.5 (SD = 11.5%) in dyadic social contexts.

**Figure 2:**
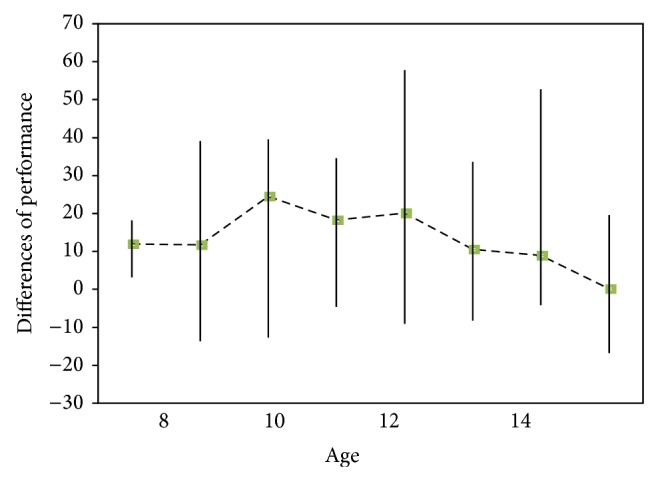
Differences of performance in monadic and dyadic contexts to show the effect of context on biological motion detection across age.

To examine to what extent differences of performance in dyadic versus monadic contexts were predicted by independent factors, we conducted a linear regression on mean differences of action perception. Results showed that systemizing ability (*t* = −3.42, *P* = 0.001) and age (*t* = −2.18, *P* = 0.03) were both negative independent predictors of differences in performances. In other words, differences of action perception between dyadic and monadic contexts were larger in participants with a lower systemizing ability and also in younger children. Empathizing ability and other variables were removed from the model. The mean differences of performance in action perception in two contexts (dyads – monads) related to age were presented in Figure 2.

## 4. Discussion

Review of the literature revealed mixed results in respect to development of action perception linked to context properties. Although PLDs have been used more frequently in recent investigations of children's action perception, there was still little discussion about their possible associated factors such as cognitive styles on perception of goal-directed actions. The current study was conducted in order to examine age as well as empathizing and systemizing effects on action perception in different social contexts among children. Our findings indicated that as children get older, performance in both monadic and dyadic contexts improves. However, participants generally performed better in movies that represented dyads than in movies that represented monads. Also as a novel finding, current study showed that empathizing ability was a strong predictor of performance in action perception in monadic and dyadic contexts, while systemizing could predict rate of accurate response only in monadic context.

From the study, a notable finding was that the cognitive style might have an important contribution to action perception through PLDs. There was a positive correlation between action perception in both monadic and dyadic social contexts with empathizing scores but that was different for systemizing scores. Based on* E-S* theory, empathizers outperform systemizers in terms of mind reading which is the prerequisite of higher social performance [23, 33]. Alaerts et al. [3] showed that people with higher empathizing abilities were more competent in intention reading of goal-directed actions through motion cues. Thus we assume that empathizing ability strongly contributes to action perception of social PLDs among participants regardless of context.

On the other side although results showed that systemizing score was not correlated with action perception in dyadic context, it was associated with participants' performance in monad PLDs. It has not been previously discussed; however, a possible explanation may be that perception of noninterpersonal activities (monads) recruits distinct mechanisms compared with interpersonal actions (dyads). It was documented that higher systemizing ability resulted in better performance in form perception tests (e.g., embedded figure task) [23, 24, 30, 34]. The monadic context may primarily require the individual to understand the form or structure of an object rather than other components. Since systemizers show higher ability in recognizing the patterns in an object-related context, one can argue that systemizing ability may help children to perform better in specific contexts with higher ratio of object/person [23, 35–37].

In line with previous research, present findings acknowledged that action perception of biological motions improves with age [38]. Neuroanatomical research has shown that improvement in both bottom-up visual inputs (e.g., maturation of ventral and dorsal visual streams) [39] and top-down cognitive mentalization outputs (e.g., increased activation of STS) occurs when children become older. These changes of brain activity may lie beneath the development of action perception across the age [7, 40]. Furthermore, one can explain that forming an internal repertoire which is related to an action would be facilitated with age. Hence, with cognitive progress, children are able to improve the capacity of mastering skills in action perception and intention reading [41–44]. In other words, as children grow up, they will have more opportunities to socialize and interact with biological agents (e.g., other humans) as well as nonbiological agents (e.g., objects) which result in obtaining valuable experiences in different social contexts.

The current study found that as age goes up, children have more similar performance in dyadic and monadic contexts. Social context properties (e.g., number of social cures) may be more critical for action perception in early childhood than in later years. This can be due to the improvement in theory of mind ability which assists children to increase their abilities in attribution of mental states regardless of context. Looking at Figure 2, findings indicated that differences in action perception increased from age 8 to 10; this finding corroborates the idea of Carter and Pelphrey [7] who demonstrated that differences of STS activation increase in the contexts with biological motion against nonbiological motion stimuli among children aged 8 to 10. Never before discussed, our findings indicated that the difference started to decrease after age of 10. Current study added to the literature that the role of context in rate of accurate action perception may be decreased in endpoint of childhood. Furthermore it could conceivably be hypothesized that the recognition of dyadic and monadic actions follows the different developmental trajectories.

The results of current study were also consistent with previous studies which found that individuals make better scores in dyadic than monadic PLDs [6, 9, 10]. Whereas a dyadic context (person-person interaction) consisted of more biological cues than a monadic situation (person-object interaction), it could be assumed that visual sensitivity and attention to the social cues would increase in dyadic contexts [6, 9]. In other words, accurate rate of performance in each context may be due to the ratio of person/object cues in that social context. Certainly, current findings are not enough to elucidate the mechanisms underlying better performance in dyadic than monadic PLDs; thus, examining further context properties (such as level of difficulty) and participants' characters (such as basic sociocognitive abilities) would be helpful.

### 4.1. Limitation and Future Directions

However these findings are limited by the use of a cross-sectional design and thus prospective studies are required to obtain a robust interpretation of action perception development. Furthermore, it should be seen as preliminary findings since the current study has only examined boys in a narrow age range. Besides, only profile or side view of actions was used in this study and potentials of other view angles were neglected. Although examining a full cognitive profile of participants was beyond the scope of this study, future research is warranted to investigate the role of other cognitive factors such as executive function skills in parallel with cognitive styles in action perception. Furthermore, this research is served as a base for future imaging studies examining neural networks linked to action perception in different social contexts (dyads or monads). Additionally, it is suggested to investigate the effects of physical properties of actions such as form, speed, size, and distance which can elucidate the mechanisms underlying action perception through children's development.

## 5. Conclusion

Our findings showed that young people perform better in dyadic against monadic social contexts though action perception in these two distinct conditions may develop differently across childhood and adolescence. Furthermore, cognitive style is a determinant factor of PLDs action perception; children with higher empathizing ability may show better performance in both monadic and dyadic contexts but systemizing contributes to performance in monadic PLDs.

## Figures and Tables

**Table 1:** Predictors of performance in dyadic and monadic social contexts.

	Unstandardized coefficient (*B*)	Standardized coefficient (*β*)	*t *	*P*
Monadic context performance				
Empathizing	0.54	0.33	4.57	<0.001
Age	3.89	0.40	5.92	<0.001
Systemizing	0.24	0.17	2.34	0.02
Dyadic context performance				
Age	2.233	0.32	4.32	<0.001
Empathizing	0.36	0.31	4.13	<0.001
